# The phylogenetic position of a new species of *Plakobranchus* from West Papua, Indonesia (Mollusca, Opisthobranchia, Sacoglossa)

**DOI:** 10.3897/zookeys.594.5954

**Published:** 2016-05-30

**Authors:** María Angélica Meyers-Muñoz, Gerard van der Velde, Sancia E.T. van der Meij, Bart E.M.W. Stoffels, Theo van Alen, Yosephine Tuti, Bert W. Hoeksema

**Affiliations:** 1 Radboud University Nijmegen, Institute for Water and Wetland Research, Department of Animal Ecology and Physiology, P.O. Box 9010, 6500 GL Nijmegen, The Netherlands; 2 Naturalis Biodiversity Center, Darwinweg 2, 2333 CR Leiden, The Netherlands; 3 Oxford University Museum of Natural History, Parks Road, Oxford OX1 3PW, United Kingdom; 4 Radboud University Nijmegen, Institute for Water and Wetland Research, Department of Microbiology, P.O. Box 9010, 6500 GL Nijmegen, The Netherlands; 5 Research Centre for Oceanography (RCO), Indonesian Institute of Sciences (LIPI), Jl. Pasir Putih I, Ancol Timur, Jakarta 14430, Indonesia

**Keywords:** COI, phylogeny, Plakobranchus ocellatus, reproductive system, Sacoglossa, taxonomy

## Abstract

*Plakobranchus papua* Meyers-Muñoz & van der Velde, **sp. n.** from West Papua (Papua Barat province, Indonesia), is described based on its external morphology, colour pattern, internal anatomy, radula and reproductive system. In a molecular phylogenetic study specimens of this new species were compared with those of ten candidate taxa under the name *Plakobranchus ocellatus* van Hasselt, 1824. DNA analyses of COI mtDNA showed a clear distinction between *Plakobranchus papua* **sp. n.** and “*Plakobranchus ocellatus*”. *Plakobranchus papua*, **sp. n.** also differed from all taxa that have been synonymised with *Plakobranchus ocellatus*. The genus is in dire need of taxonomic revision, preferably based on an integrative analysis involving morphology and DNA of all known *Plakobranchus* varieties.

## Introduction

Sea slugs of the genus *Plakobranchus* van Hasselt, 1824 (Order Sacoglossa, Suborder Plakobranchacea) have an elongated body and dorsoventrally flattened, lateral parapodia, which are folded up on the dorsal surface (van Hasselt 1824; Jensen 1992). According to Jensen (1997a) this genus possesses a number of plesiomorphic characters such as an anterodorsal anus, a pharyngeal pouch, triangular, denticulate teeth, a long, curved penial stylet, and the absence of dorsal vessels. The genus also possesses a number of autapomorphies: a broad and flat head, rhinophores located at the anterior corners, mediodorsal eyes on a small papilla, numerous longitudinal dorsal lamellae containing branches of the digestive gland, and a truncate tail; the hermaphrodite ampulla has apparently been lost.

These animals can be found in shallow sandy habitats, crawling over it or half-buried (Gosliner et al. 2008; Mehrotra et al. 2015), or on coral rubble and in rock pools (Yonow 2008) where they consume green macroalgae (Jensen 1993).

*Plakobranchus ocellatus* van Hasselt, 1824, feeds on a wide variety of marine green algae (Chlorophyta), including at least five species of Ulvophyceae (Wägele et al. 2011). Many studies on *Plakobranchus* deal with their kleptoplasty, the ability to retain functional chloroplasts from their green algae in their digestive gland cells (Clark et al. 1990; Jensen 1996, 1997a). Species of this genus as well as other sacoglossans belong to the few known animal species with the ability of photosynthesis (Trench 1969; Hirose 2005; Bass 2006; Händeler et al. 2009; Maeda et al. 2010; Wägele et al. 2011; Christa et al. 2013; Yamamoto et al. 2013).

*Plakobranchus* species are simultaneous hermaphrodites, which possess a penial stylet used in hypodermic insemination. Penial stylets and hypodermic insemination are commonly found within the Sacoglossa (Schmitt et al. 2007; Smolensky et al. 2009). Jensen (1992) also observed extensively branched prostate and albumen glands and a pair of secondary copulatory bursae in *Plakobranchus*, which are unique for the genus.

**Figure 1.**
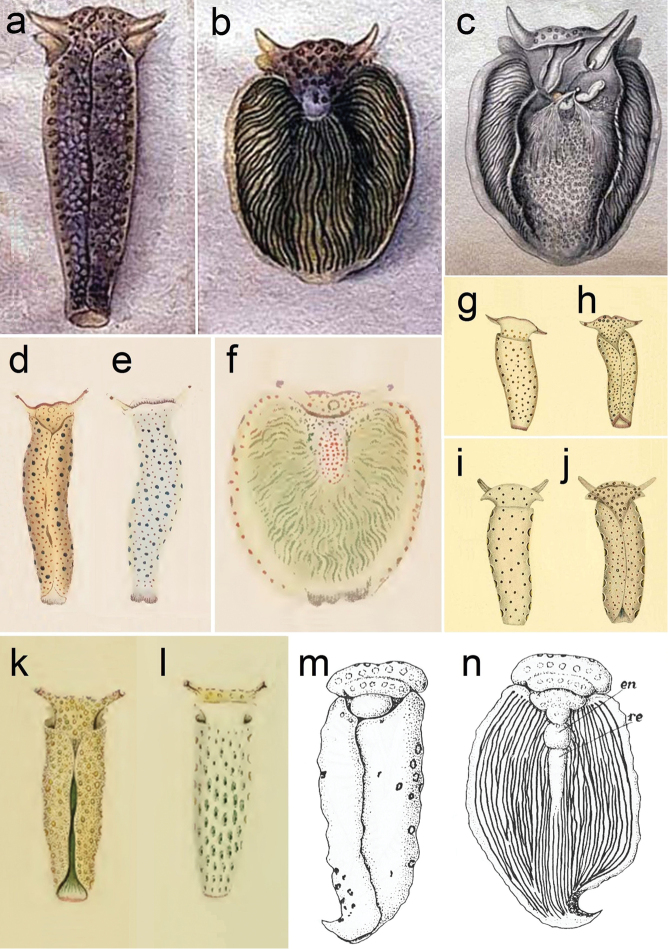
**a–c** *Plakobranchus ocellatus*, drawings by van Hasselt (1824): **a** dorsal view with parapodia folded up on the dorsal body surface **b** dorsal view with open parapodia, showing longitudinal lamellae **c** internal anatomy **d–f** *Placobranchus ianthobaptus*, drawings by Gould (1852): **d** dorsal view with parapodia folded up on the dorsal body surface **e** ventral view **f** dorsal view with open parapodia, showing longitudinal lamellae **g–j** two *Plakobranchus* species illustrated by Pease (1871) **g–h** *Placobranchus gracilis*: **g** ventral view **h** dorsal view **i–j** *Placobranchus variegatus*: **i** ventral view **j** dorsal view **k–l** drawings of *Plakobranchus chlorophacus* by Bergh (1873): **k** dorsal view with parapodia folded up on the dorsal body surface **l** ventral view with ocellated spots **m–n** drawings of *Plakobranchus ocellatus* by Marcus (1982): **m** dorsal view with parapodia folded up on the dorsal body surface **n** dorsal view with open parapodia, showing the longitudinal lamellae (en = pericardium; re = renal prominence).

During the last decades only *Plakobranchus ocellatus* has been considered a valid species within the genus (Jensen 1992). This species was described from shallow waters in the Sunda Strait near Anyer, northwest Java, Indonesia. van Hasselt (1824) gave this name because of the blue- and yellow-centred ocellated spots covering the dorsal side and flanks of the body (Figure 1). The original description is based on the species’ phenotype and some characteristics of the parapodial lamellae, heart and reproductive system as shown in the original illustrations supplied by van Hasselt (1824). The name *Plakobranchus* has occasionally been misspelled as *Placobranchus*, which started when the original description was translated from Dutch to French in 1824 (Bergh 1887; Jensen 1997a, 1997c).

Jensen (1992) studied the anatomy of several *Plakobranchus* specimens from various Indo-West Pacific locations (Red Sea, Thailand, Guam) and synonymised the previously described species. The descriptions were mainly based on external anatomy, colour pattern and geographic distribution (Yonow 1990, 2008; Debelius 1996; Jensen 1997a; Marshall and Willan 1999; Gosliner et al. 2008) and hardly included descriptions of the internal anatomy as presented by van Hasselt (1824) and Jensen (1992). *Plakobranchus ocellatus* is now considered the only known valid species of the genus, with ten synonyms and a wide Indo-Pacific distribution (Jensen 2007). This may have been premature (Jensen 1992, 1997a, 1997c), because Gosliner et al. (2008) distinguished two undescribed *Plakobranchus* species in addition to *Plakobranchus ocellatus*. Subsequently, Krug et al. (2013), who used the mitochondrial barcoding gene COI and the nuclear histone 3 gene, found ten distinct phylogenetic lineages in *Plakobranchus*. This suggests that the taxonomy of *Plakobranchus* still deserves further study. In the present study a new *Plakobranchus* species from West Papua, Indonesia, is described and a phylogenetic reconstruction based on the mitochondrial barcoding COI gene is included to show its position within the genus *Plakobranchus*.

## Material and methods

**Figure 2.**
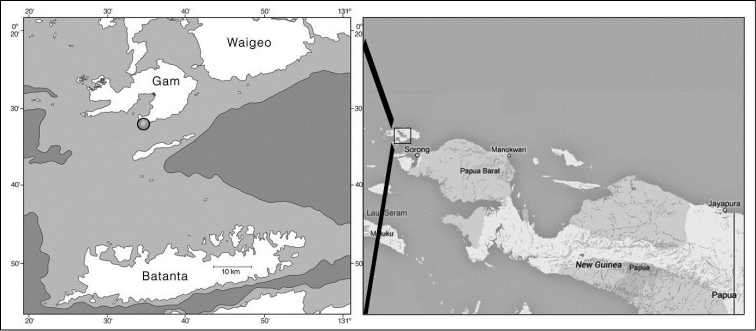
Map of West Papua and Papua, Indonesia, showing the area and locality where *Plakobranchus papua* specimens were collected: south Gam Island, Mangrove Creek.

Twenty specimens were collected by Gerard van der Velde in Indonesia during the 2007 Raja Ampat Expedition (Figure 2; Hoeksema and van der Meij 2008). The specimens were observed alive, photographed, and subsequently preserved in 96% ethanol. Material analysed in this study was deposited in the mollusc collection of Naturalis Biodiversity Center, Leiden, The Netherlands, and catalogued as RMNH.MOL. One specimen of *Plakobranchus ocellatus* (RMNH.MOL.336426), collected in the Philippines, 4 November 1999 (Sta. CEB.01, Cebu Strait, E side of Olango Island 10°15'54"N 124°04'17"E, coll. BWH) was used for comparison of external characters with the new species in absence of the holotype of *Plakobranchus ocellatus* (Figures 7b–d). The original drawings of van Hasselt (1824) are available in scientific archives of Naturalis Biodiversity Center and reprinted here (Figures 1a–c).

To study the radula, the buccal masses of two specimens were dissected and immersed in 10% NaOH until the tissue surrounding the radulae was dissolved. The radulae were rinsed in distilled water and transferred to 70% ethanol. They were subsequently examined by means of a light microscope, photographed, mounted on stubs, and gold-sputter-coated for scanning electron microscopy (SEM). Eight specimens were dissected for anatomical studies. One specimen (Table 1) was dehydrated in a graded ethanol series, embedded in paraffin, and cut into 7 μm serial sections with a manual microtome for histological observations. Sections were stained with toluidine blue to identify structures on the basis of metachromasia.

**Table 1.** COI data from Plakobranchus cf. ocellatus from GenBank, for comparison with the sequence data of *Plakobranchus papua* sp. n.

Species	Accession number	Publication	Collection locality
Plakobranchus cf. ocellatus	AB501307	Maeda et al. 2010	Okinawa, Japan
	AB758968-971	Takano et al. 2013	Okinawa, Japan
	DQ237996	Klussmann-Kolb and Dinapoli 2006	Eastern Australia
	DQ471269	Bass unpubl.	Guam
	DQ471270	Bass unpubl.	Hawaii
	GQ996679-680	Händeler et al. 2009	Eastern Australia
	JX272685-720	Christa et al. 2013	Philippines
	KC573714-715	Krug et al. 2013	Okinawa, Japan
	KC573716	Krug et al. 2013	Vanuatu
	KC573717	Krug et al. 2013	Guam
	KC573718-719	Krug et al. 2013	Okinawa, Japan
	KC573720	Krug et al. 2013	Philippines
	KC573721-722	Krug et al. 2013	Guam
	KC573723-724	Krug et al. 2013	Andaman Sea, Thailand
	KC573725	Krug et al. 2013	Eastern Australia
	KC573726-727	Krug et al. 2013	Okinawa, Japan
	KC573728-729	Krug et al. 2013	Moorea, French Polynesia
	KC573730	Krug et al. 2013	Guam
	KC573731	Krug et al. 2013	Okinawa, Japan
	KC573732	Krug et al. 2013	Sulawesi, Indonesia
	KC573733	Krug et al. 2013	Philippines
	KC573734	Krug et al. 2013	Papua New Guinea
	KC573735-737	Krug et al. 2013	Philippines
	KC573738	Krug et al. 2013	Hawaii
	KC706898	Leray et al. 2013	French Polynesia
	HM187633-634	Wägele et al. 2011	Guam
	HM187635	Wägele et al. unpubl.	Guam
	HM187638	Wägele et al. 2011	Guam
*Elysia ornata*	AB758962	Takano et al. 2013	Japan
*Thuridilla carlsoni*	GQ996681	Händeler et al. 2009	Eastern Australia

The holotype (RMNH.MOL.336417) and two paratypes (RMNH.MOL.336418–336419) of *Plakobranchus papua* sp. n. were used for DNA analysis. A piece of foot tissue was extracted and treated according the DNeasy Blood and Tissue, spin-column protocol for the purification of total DNA. Primers (LCOI490–HCO2198) were used for the amplification of the mitochondrial gene COI (cytochrome c oxidase subunit, partial) region (Folmer et al. 1994).

PCR reactions were performed with Thermocycle Biometra T professional. All PCR reactions were carried out in 50 μl reaction volume, including 25 μl Q PerfeCTa®SYBR® Green FastMix® from Quanta BioScience Inc, (Gaithersburg, USA), 2 μl of each primer, 19μl of DPEC treated water and 2 μl of genomic DNA. Thermal cycling started with an initial melting step for 3 min at 94 °C, followed by 39 cycles at 94 °C for 15 sec, 50 °C for 30 sec, annealing using a temperature gradient from 50–60 °C for 1 min, 72 °C for 1 min, and a final elongation step at 72 °C for 5 min.

The PCR products were purified with a Gene JetTM PCR Purification kit (Fermentas Life Sciences Lithuania) and sequenced directly, using the same primers as for PCR. DNA sequences were obtained using the Big Dye terminator ver. 3.1 Cycle Sequencing kit (Applied Biosystems) and analysed with the automated sequencer ABI 3130 Genetic analyzer at the sequencing facility of the Department of Anthropogenetics at the University Medical Centre, Radboud University, Nijmegen. The sequences were analysed with the program Chromas Lite (Technelysium Pty Ltd.) and deposited in Genbank (KU934191–KU934193).

Phylogenetic analyses were carried out on a dataset of 81 sequences including two outgroup species (Table 1). All COI sequences of *Plakobranchus* available on GenBank were used, and three newly obtained sequences of *Plakobranchus papua* sp. n. were added. *Elysia ornata* (Swainson, 1840) (AB758962) and *Thuridilla carlsoni* Gosliner, 1995 (GQ996681) were selected as outgroups (Bass and Karl 2006). Sequences were aligned using the Guidance server (ClustalW), resulting in an alignment score of 1.00 for the dataset (Penn et al. 2010). A model selection analysis was carried out in jModeltest (Posada 2008) to select the best-fit model based on AICc (corrected Akaike Information Criterion), rendering TrN + G as the best-fit model. A maximum likelihood analysis using the GTR + G model (1000 bootstraps) was carried out in Phyml 3.1 (Guindon et al. 2010) in the Seaview platform (Gouy et al. 2010) and a majority rule consensus tree was constructed. Bayesian inferences (3,000,000 million generations) were estimated in MrBayes 3.1.2 (Ronquist and Huelsenbeck 2003) using the GTR + G model (because of unavailability of the TrN + G model). Average standard deviation of split frequencies was 0.09851. A majority-rule consensus tree was constructed in MrBayes with a burnin of 25%, and visualised in FigTree 1.3.1 (http://tree.bio.ed.ac.uk/software/figtree/).

The web version of ABGD (Automatic Barcode Gap Discovery, Puillandre et al. 2012) was used to estimate the genetic distance corresponding to the difference between a speciation process versus intraspecific variation. Runs were performed using the default range of priors (pmin = 0.001, pmax = 0.10) using the JC69 Jukes-Cantor measure of distance. The analysis involved 25 nucleotide sequences. All ambiguous positions were removed for each sequence pair; there was a total of 657 positions in the final dataset.

## Systematics

### Suborder Plakobranchacea Gray, 1840 Superfamily Plakobranchoidea Gray, 1840 Family Plakobranchidae Rang, 1829

#### Genus Plakobranchus

Taxon classificationAnimaliaSacoglossaPlakobranchidae

van Hasselt, 1824

##### Genus diagnosis

(emended after Jensen 1992). Genus characterized by a truncate and flattened body shape, flat head, and enrolled rhinophores. Small admedian eyes. Parapodia folded up on the dorsal body surface, parapodial lamellae containing branches of the digestive gland, dorsal vessels absent. Anterodorsal anus. Long curved penial stylet.

#### Type species: *Plakobranchus ocellatus* van Hasselt, 1824

Figures 7b–e

**Figure 3.**
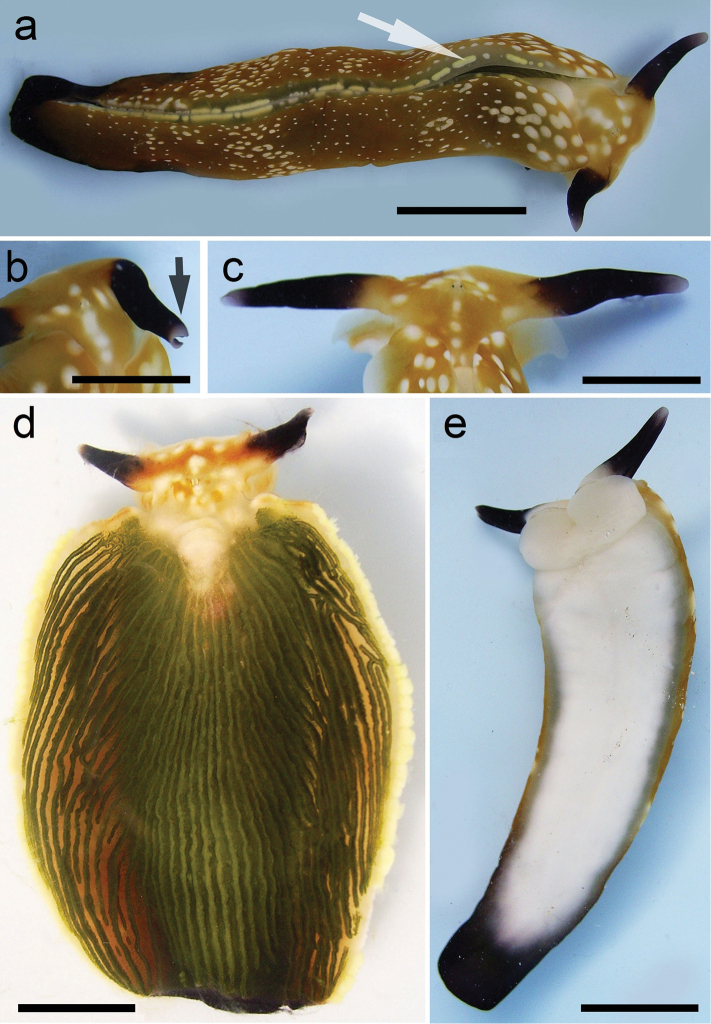
Plakobranchus papua. Images taken from live animals (external morphology). **a** Dorsal view, parapodia folded up on the dorsal body surface; arrow shows short yellow rod-like spots along the parapodial border **b** Dorsal view with detail of the rolled rhinophore indicated by an arrow **c** Detail of head, rhinophores, and pedal tentacles **d** Open parapodia with lamellae containing branches of the digestive gland and showing renopericardial area **e** Ventral view of bilobed oral prominence, narrow foot, and truncated black tail. Scale bars: **a**, **d**, **e** = 10 mm; **b**, **c** = 5 mm

**Figure 4.**
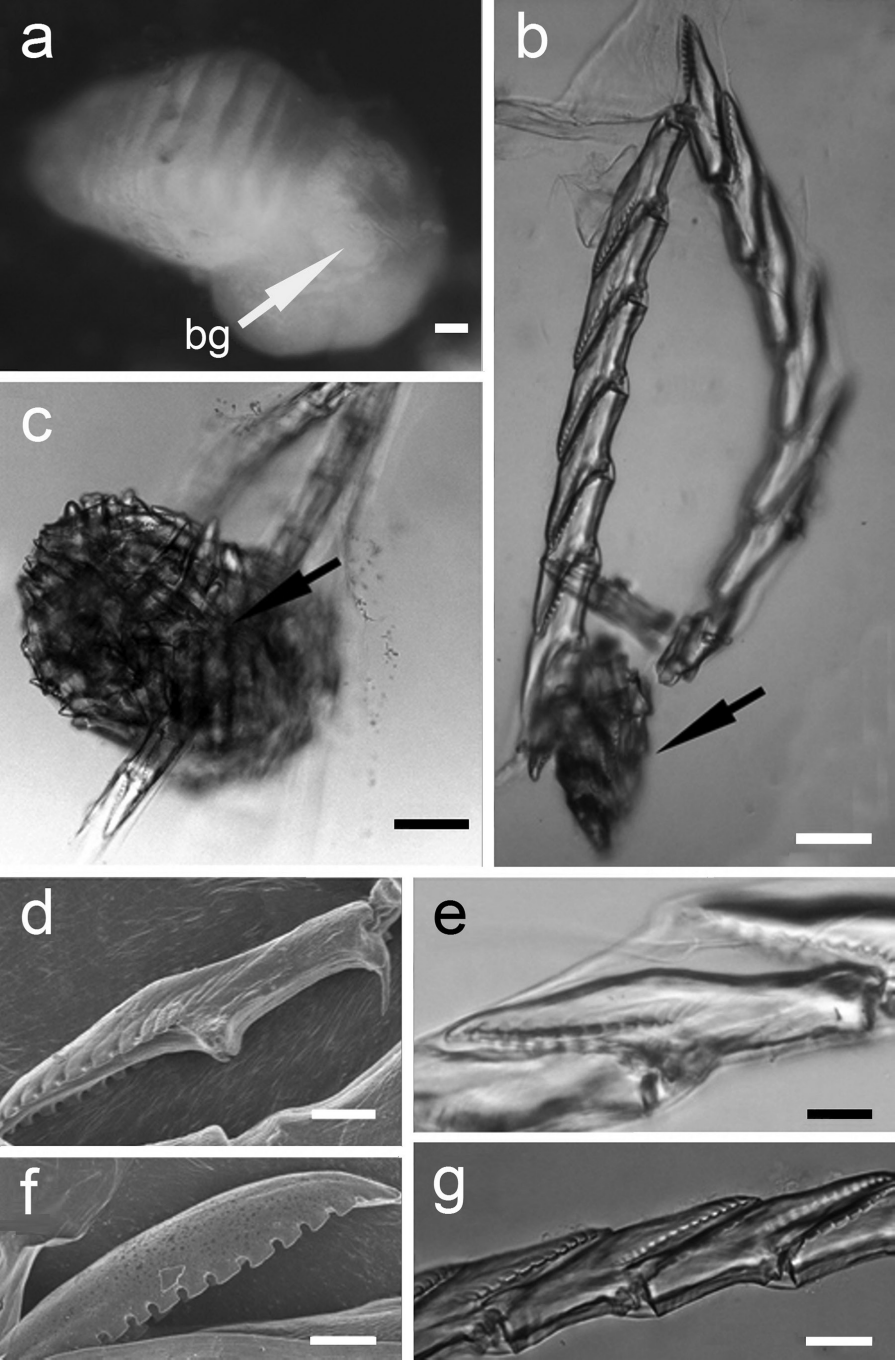
Plakobranchus papua. Masticatory apparatus. **a** Pharynx; white arrow points to the buccal ganglion (bg) **b** Radula with ascus-sac (arrowed). **c** Detail of the ascus-sac which contain used teeth (arrowed) **d** Tooth, scanning electronic photograph **e** Tooth, light microscopy photograph **f** Detail of the denticles (SEM) **g** Row of teeth (LM). Scale bars: **a, c** = 50 μm; **b** = 25 μm; **d–e** = 10 μm; **f** = 5 μm; **g** = 15 μm.

**Figure 5.**
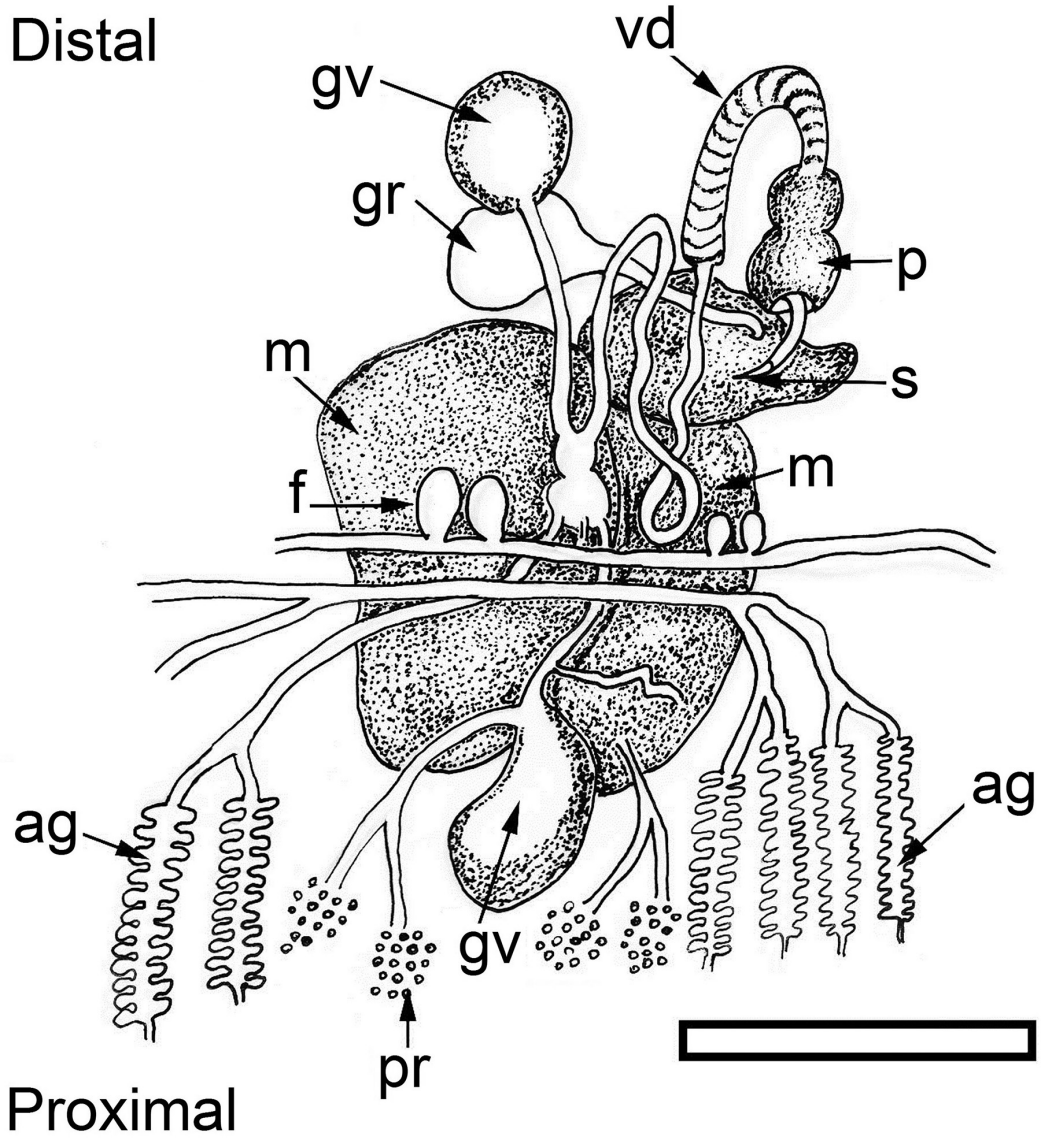
Plakobranchus papua. Schematic drawing of reproductive system (terminology after Jensen 1992). Scale bar: 250 μm. Abbreviations: ag = albumen gland, f = follicles, gr = genital receptacle, gv = gametolytic vesicle (‘bursa copulatrix’), m = mucus gland, p = penial bulb, pr = prostate, s = penial stylet, vd = vas deferens.

**Figure 6.**
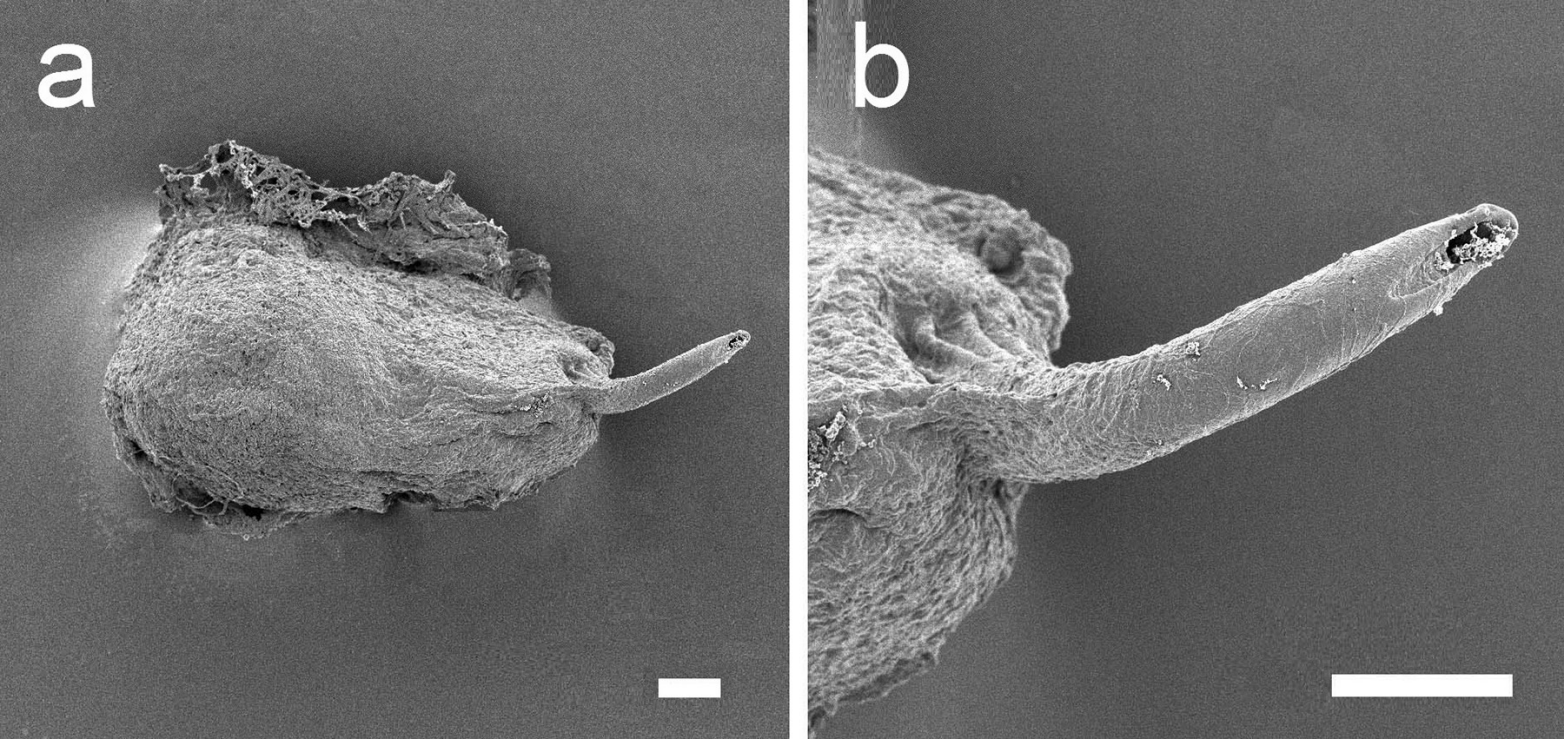
Plakobranchus papua. **a** Muscular penial bulb and stylet **b** Detail of the opening of the stylet. Scale bars: 100 μm.

**Figure 7.**
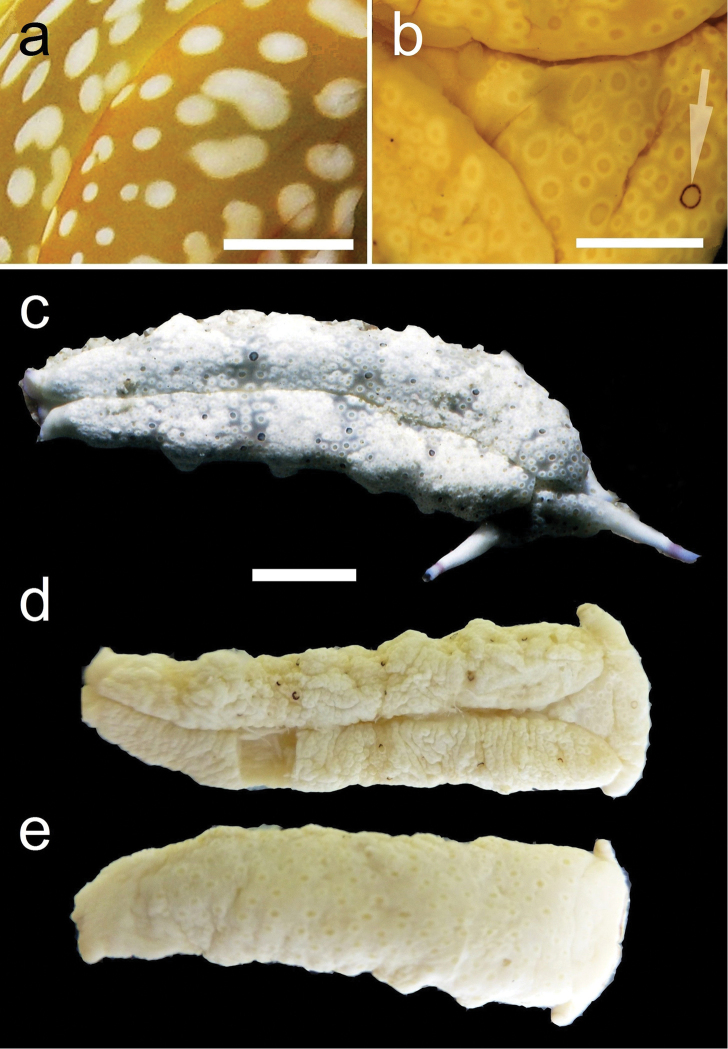
**a** Dorsal view of a preserved Plakobranchus papua showing white dots surrounded by a yellow outline. **b** Preserved Plakobranchus ocellatus, ocellated spots with dark pigment surrounded by a white ring, some with a black ring (arrowed). **c** Plakobranchus ocellatus, dorsal view of a live animal (Cebu, Philippines, 1999, photo BWH). Plakobranchus ocellatus, (**d**) dorsal view and (**e**) ventral view of preserved animal. Scale bars: **a**, **b** = 5 mm, **c–e** = 10 mm.

**Figure 8.**
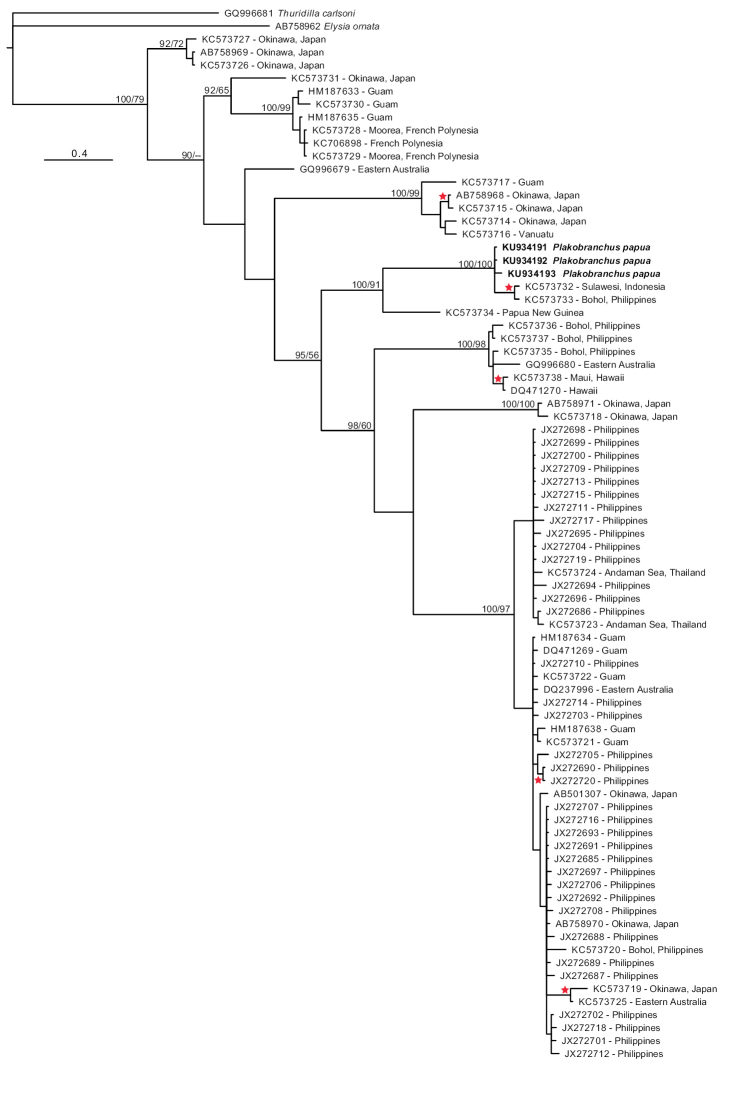
Phylogram of the *Plakobranchus ocellatus* species complex, topology derived from MrBayes analysis. Support values represent Bayesian posterior probabilities / ML. Numbers refer to GenBank codes. Red stars represent nodes with Bayesian probability values > 90 and high ML values.

*Plakobranchus ocellatus* van Hasselt 1824: 34–36 (near Anyer, Serang, Bantam, Indonesia; holotype lost); Cuvier 1830: 56; ed. Mason Moll.: 84, pl. 30, figs 7, 7a; Quoy and Gaimard 1833: 319; Bergh 1872: 147–151, pl. XIX figs 1–13; Bergh 1873: 75-76; Vayssière 1912: 111–112, pl. 1 fig. 9; Baba 1936: 19–20; Burn 1972: 177, fig 11; Kay 1979: 454, fig. 144F; Bertsch and Johnson 1981: 20–21; Marcus 1982: 17–18, figs 35–38; Yonow 1990: 288, pl. 2; Colin and Arneson 1995: 176–177, figs 811–812; Debelius 1996: 159, 170; Erhardt and Baensch 1998: 634, 668; Marshall and Willan 1999: 37–38, fig. 48; Coleman 2001: 131; Hirose 2005: 905–916, figs 1–29; Jensen 2007: 278; Coleman 2008: 88–89; Yonow 2008: 56, 124–125, fig: 47; Gosliner et al. 2008: 93–94; Apte 2009: 169, fig. 2a; Hervé 2010: 115; Ramakrishna et al. 2010: 53–54, figs 31, 31 (two images with same number); Yonow 2012: 20, pl. 17; Trowbridge et al. 2011: 2, 6, 8; Sreeraj et al. 2012: 2501.

*Placobranchus ianthobaptus* Gould 1852: 307, pl. 26, figs 407a–c (Honolulu, Hawaii); Bergh 1872: 166; Vayssière 1912: 47; Ostergaard 1955: 120–122, fig. 8a–f.

*Placobranchus guttatus* Stimpson 1855: 378–379 (Loo Choo Is.); Trowbridge et al. 2011: 2.

*Elysia ocellata* Pease 1860: 35 (Sandwich Island, Honolulu).

*Placobranchus gracilis* Pease 1871: 303, pl. 21, figs 1a–b (Tahiti, French Polynesia); Bergh 1872: 166.

*Placobranchus variegatus* Pease 1871: 303–304, pl. 21, figs 2a–b (Huaheine, French Polynesia); Bergh 1872: 166–167.

*Plakobranchus argus* Bergh 1872: 151–165, pls. IX figs 6–9, XVII, XVIII (Honolulu, Hawaii); Vayssière 1912: 111.

*Plakobranchus camiguinus* Bergh 1872: 167–169, pl. XIX, figs 14–19 (Luzon, Philippines).

*Plakobranchus laetus* Bergh 1872: 171–173, pl. XIX, figs 28–31, pl. XX, figs 1–7 (Masoloc, Philippines).

*Plakobranchus priapinus* Bergh 1872: 173–174, pl. XVIII, figs 17–18, pl. XX, figs 8–13 (Bohol, Philippines); Bergh 1905: 81–82, pl. II, fig. 21, pl. XIII, figs 18–19.

*Plakobranchus punctulatus* ? Bergh 1872: 169–171, pl. IX, figs 11–12, pl. XIX, figs 20–27 (Masoloc, Philippines); Bergh 1905: 82.

*Plakobranchus chlorophacus* Bergh 1873: 76–77 (148–149), pl. IX, fig. 5–6, pl. X, fig. 22–25, pl. XI, figs 3–6 (Huaheine, French Polynesia); Barash and Zeniper 1994: 7.

*Placobranchus ocellatus* Bergh 1887: 310–311, pl. 6, fig. 5; O’Donoghue 1928: 714; Rao 1962: 253–254, figs 1, 2e, f; Heller and Thompson 1983: 332–334, figs 6A–C; Hughes 1977: 92; Jensen 1992: 283–285, figs 22B, 23, 24D–E; Richmond 1997: 266–267.

*Plakobranchus* sp. Bergh 1905: 82–83 (Kur I., Indonesia); Trowbridge et al. 2011: 3.

#### Plakobranchus papua

Taxon classificationAnimaliaSacoglossaPlakobranchidae

Meyers-Muñoz & van der Velde sp. n.

http://zoobank.org/E6FB98EC-AD98-4675-9FC4-0E205A6E3A2F

Figures 2 , 3 , 4 , 5 , 6 , 7a

##### Type material.

Holotype RMNH MOL.336417, length 35 mm (COI, anatomy. Genbank Accession number: KU934191). Paratype RMNH MOL.336418, length 30 mm (COI, anatomy, radula. Genbank Accession number: KU934192). Paratype RMNH MOL.336419, length 34 mm (COI, anatomy. Genbank Accession number: KU934193).

##### Additional material.

RMNH MOL.336420, length 28 mm (anatomy, radula). RMNH MOL.336421, length 25 mm (anatomy, radula, penial bulb). RMNH MOL.5010422–5010434, slides of a single specimen, length 30 mm, northeast of Pulau Mansuar (S00°34.083', E130°38.525), Expedition Sta. RAJ.38, 30 November 2007 (histology). RMNH MOL.336423 (n = 7), length 15–26 mm, east side of Pulau Kri, Sorido Resort lagoon, near Jetty (S00°33.347', E130°41.225), Expedition Sta. RAJ.02, 4 December 2007 (anatomy, penial bulb). RMNH MOL.336424, length 41 mm, south side of Pulau Kri (S00°33.536', E130°41.258'), Expedition Sta. RAJ.03, 4 December 2007 (anatomy, penial bulb). RMNH MOL.336425 (n = 6), length 15–26 mm, west side of Pulau Yeben Kecil (S00°29.344', E130°30.081'), Expedition Sta. RAJ.48, 6 December 2007 (anatomy, radula).

##### Type locality.

Mangrove Creek, south Gam Island (0°30'403"S, 130°38'986"E), West Papua, Indonesia, 25 November 2007.

##### Habitat.

The specimens were collected in sea grass beds and on coral reef sand between 1 and 10 m depth.

##### Etymology.

The specific name *papua* of this species is based on the name of the Indonesian part of New Guinea (provinces Papua and West Papua) where the type material was collected.

##### Description.

*External morphology* (Figure 3). Body elongated, dorsoventrally flattened with wide parapodial flaps folding along the dorsal midline (Figure 3a). Rhinophores extended from lateral edges of the head long, smooth, rolled (Figure 3b). An eye pair belonging to the central nervous system (CNS) is visible on the head. Eyes situated very close to each other, in front of a prominent, elongated white spot (Figure 3c). The voluminous renopericardial prominence is short, whitish (Figure 3d), and covered by the parapodial flaps. The folded parapodia show a smooth surface from a dorsal view. The parapodia margin at the mid-line shows short yellow rod-like spots along the complete parapodial border (Figure 3a) such as those visible in ‘*Plakobranchus ocellatus*’ (Wägele et al. 2011: fig. 1a). When the parapodia are open, the edges appear as soft yellow pectinate margins (Figure 3d). Internally, the parapodia have thick parallel longitudinal lamellae. Only the mid-central lamellae connect directly to the renopericardial prominence and run parallel to each other towards the posterior side of the body. The most external anterior lamellae are shorter, connected through anastomosing lamellae with the most internal lamellae and through them connected with the renopericardial prominence (Figure 3d). These fine dorsal anastomosing lamellae can only be recognized in live and in freshly collected, non-preserved specimens. In preserved contracted specimens it is not possible to distinguish these dorsal anastomosing lamellae. The lamellae possess visible internal granulations corresponding to the digestive gland ramifications, which contain chloroplasts. The genital opening is situated on the right anterior side of the body, just in front of the anterior part of the parapodia, and immediately behind the rhinophore. The anal opening is located on the right anterior side of the pericardium. Ventrally a bilobed oral prominence (Figure 3e) with a very fine, undulating black line boarding the upper lip is present. Pedal tentacles are short (Figure 3c). The narrow foot has a smooth surface and truncated tail (Figure 3e).

*Colouration* (Figure 3). Intense ochre body with white spots scattered all over the dorsum, head, and flanks: closest to the head region, on the anterior side corresponding to 1/4 of the body length, the spots are largest, on 2/4 and 4/4 of the body length the dots are smaller, and on the 3/4 of the body length they are larger, but not as large as on the anterior part of the body. Spots are pale white with a thin yellow outline (Figure 7a). Rhinophores black, the outer tips purplish. Internal parapodial flaps ridges bright green to olive green in colour, owing to chloroplasts in the digestive gland. The pericardium region is hyaline white. The pedal tentacles are translucent white. The foot sole is white with a black tail.

*Digestive system* (Figure 4). The masticatory apparatus was studied in four preserved specimens (RMNH.MOL.336418, 336420, 336421, 336425). The pharynx is connected to the stomach through a short muscular lightly bent oesophagus. The stomach is approximately 25% smaller than the pharynx. A pair of salivary glands inserted and extended along the oesophagus, reaching the first third of the stomach. The pharynx has prominent dorsal septate muscles. It consists of a large cuticular pharyngeal cavity, the radular sac, and one large ascus, which are also embedded in cuticular material. Uniserial radula, small, approximately 0.50 to 0.75 mm in length with 15 teeth, eight in the ascending series and seven in the descending series (Figure 4b), and an ascus-sac which varies in size (Figures 4b, c). Teeth are sharp with triangular cusps and 10–14 denticles at each margin side, the teeth measuring 70–75 μm (Figure 4d–g).

*Reproductive system* (Figures 5–6). Occupies almost the central anterior part of the body, at nearly 1/4 of the body length. The distal part of the reproductive system is situated below the heart and directly behind the central nervous system. The penial bulb, which is only approximately 0.5 mm long (Figure 5: p), is situated below the rhinophores at the same level as the eyes. It possesses a sharp cuticular stylet, which is hollow, with an oblique orifice at the tip (Figure 6). The vas deferens (Figure 5: vd) bends over the mucus gland (Figure 5: m), and is orientated towards the proximal area. The distal part of the vas deferens, which is in direct connection with the penial bulb, is muscular and arched. Its proximal part is thin and coiled and is connected to two spherical genital vesicles (Figure 5: gv). After this intersection the vas deferens continues in a proximal direction, where it appears as a short bulky extension, continuing in a thin duct attached to numerous ramifications, the follicles. Underneath this vesicle one large piriform white genital receptacle is present (Figure 5: gr), connected directly to the mucus gland. The mucus gland is large and divided in two lobes. The distal lobe is smaller, a little narrow and coiled. The prostate (Figure 5: pr) and albumen glands (Figure 5: ag) are extensively branched. The terminology used here is similar to the one in the schematic drawing of the reproductive system of *Plakobranchus ocellatus* by Jensen (1992: fig. 22B).

*Central nervous system*. The central nervous system is located at the anterior part of the oesophagus and forms a circumoesophageal ring, consisting of a very small pair of buccal ganglia, a large pair of fused cerebro-pleural ganglia, and a pair of pedal ganglia. The CNS consists for the main part of the cerebral and pleural ganglia. The eyes of the CNS are situated very close to each other.

*DNA analyses* (Figure 8). The molecular phylogeny reconstruction of the genus *Plakobranchus* contains all the currently available sequences on GenBank. The analyses in MrBayes (Bayesian inference) and Phyml (maximum likelihood) resulted in trees in which the specimens were assigned to the same clades. In Phyml the tree formed a polytomy, whereas the analyses in MrBayes showed a topology with highly resolved clades. Ten different clades can be distinguished, of which three are represented by single specimens. The largest clade contains 49 sequences. Our new species groups with *Plakobranchus* sp. 1 of Krug et al. (2013) from Sulawesi, Indonesia and Panglao, Philippines. Over 580 base pairs there is a difference of 1.2% (7 bp) between our specimens and the specimens of *Plakobranchus* sp. 1 from Krug et al. (2013).

The ABGD analysis resulted in prior maximal intraspecific divergence of ca. 0.07. Values higher than the maximal intraspecific divergence resulted in 10 Molecular Operational Taxonomic Units (MOTUs) in both the recursive and initial partition. Each of these MOTU’s corresponds to a clade in the phylogeny reconstruction (Figure 8). The three singletons (GQ996679, KC573731, KC573734) from the ABGD analysis were also retrieved as singletons in the molecular phylogeny.

## Discussion

*Plakobranchus papua* sp. n. differs not only from *Plakobranchus ocellatus* as illustrated by van Hasselt (1824), but also from several other *Plakobranchus* colour varieties, which can be found at SeaSlugForum and NudiPixel. Colour variations of *Plakobranchus* and geographical distributions are presented in Table 2. Based on this data several species of *Plakobranchus* are to be expected. The new species differs from all other descriptions and illustrations of individuals ascribed to *Plakobranchus ocellatus* by a clearly different colour pattern, with black rhinophores and tail, non-ocellated spots, and a foot sole without spots. *Plakobranchus papua* sp. n. also differs externally from all taxa that have been synonymised with *Plakobranchus ocellatus* in the colouration, distribution of spots, and the absence of real ocellated spots. DNA analyses of COI mtDNA show a clear distinction between *Plakobranchus papua* sp. n. and *Plakobranchus ocellatus* s.l.

**Table 2.** Distribution, external morphology, and colour pattern of *Plakobranchus* varieties identified as *Plakobranchus ocellatus* and their references.

Taxon	Colour pattern	Distribution	References
*Plakobranchus ocellatus* var. A 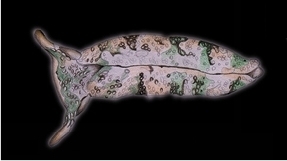	Small ocellate spots and white spots scattered over the head and dorsal flaps. Transverse dark stripes. Foot sole with some white and dark spots and some scattered ocellate spots.	Okinawa, Japan; Bai Su, Vietnam; Milne Bay and New Hanover, Papua New Guinea; Cebu, Philippines; Ambon, Indonesia	Adams 2000; Ono 2005; Krampf 2006; Gosliner et al. 2008;
*Plakobranchus ocellatus* var. B 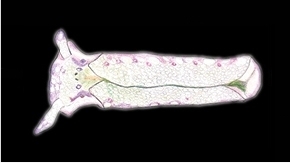	Body white cream, ocellate spots with red brown rings present over the head and lateral flanks. Dorsal white spots. Foot sole with black spots.	Okinawa, Japan	Ono 2005
*Plakobranchus ocellatus* var. C 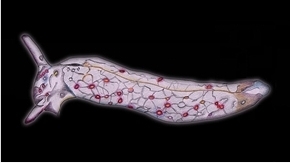	Translucent white rose body with few dark red ocellate spots over the parapodia and head. Foot sole with small dark dots green and brown.	Okinawa, Japan; Mayotte	Ono 2005; Deuss 2009
*Plakobranchus ocellatus* var. D 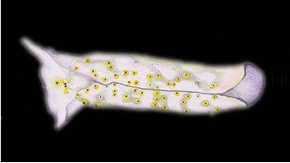	Dorsal body white-green, scattered with yellow spots. Foot sole white green, without spots.	Okinawa, Japan	Ono 2005
*Plakobranchus ocellatus* var. E 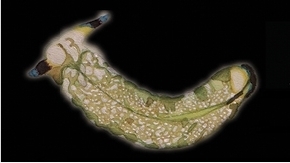	Translucent olive-green body, covered with large white-cream spots, rhinophores, oral tentacles and tail with bluish purple tips. Tail tip also black. Foot sole blue.	West Papua and Sulawesi, Indonesia; Cebu, Philippines; Palau; Milne Bay and New Hanover, Papua New Guinea; Nha Trang, Vietnam; Okinawa, Japan	Present study, Colin and Arneson 1995; Adams 2000; Warren 2000; Krampf 2006; Coleman 2008; Gosliner et al. 2008
*Plakobranchus ocellatus* var. F 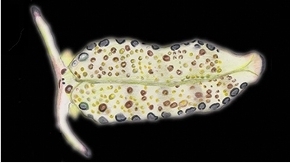	Rhinophores, oral tentacles and tail with black tips. Head and parapodia cover with many ocellate spots, small yellow and medium dark. Lateral flanks with largest black ocelli. Foot translucent green with few black ocelli.	Okinawa and Kagoshima, Japan	Imamoto 2005
*Plakobranchus ocellatus* var. G 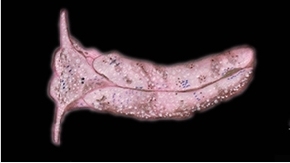	Body rose-brown, with white spots and some scattered brown and blue spots.	Moorea, French Polynesia	Geiger 2001
*Plakobranchus ocellatus* var. H 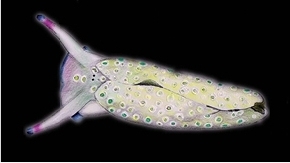	Body white-light green with a little rose on head. Yellow ocellate spots all over the parapodia, only two on head. Lateral flanks with large green ocellate spots. Oral tentacles and rhinophores tips blue. Rhinophore with a fuchsia sub-terminal ring.	Cebu, Philippines	Raabe 2006
*Plakobranchus ocellatus* var. I 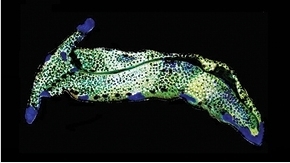	Body dark green with tiny white spots, lateral flanks, tail and rhinophores tips blue or violet, over the midline of the head with blue/violet and some scattered black spots.	Thailand; Bali and Gorontalo, Indonesia; Hawaii	Gould 1852; Coleman 2008; Gosliner et al. 2008; Supapong 2008
*Plakobranchus ocellatus* s.s. 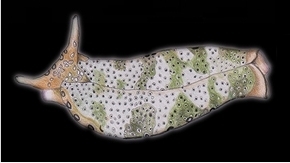	Body pale yellow-green. Head and parapodial dorsal surface covered with ocellate spots (coloured rings). Whitish, purple rhinophores, green-blue ocellata on flanks. Front of the head and foot sole with dark ocelli.	Eastern Australia; Bohol and Cebu, Philippines; Guam; Indonesia; Mayotte; Danang City, Vietnam; Red Sea; Japan; Thailand; Maui, Hawaii	van Hasselt 1824; Jensen 1992; Colin and Arneson 1995; Erhardt and Baensch 1998; Rudman 1998; Koehler 1998; Marshall and Willan 1999; Coleman 2001; Jacobson 2003; Rudman 2003; Wyatt 2003; Groeneveld 2006; Raabe 2006; Tuyen 2008; Yonow 2008; Deuss 2009
*Plakobranchus papua* sp. n. 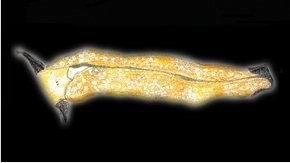	Body yellow-orange with white spots on the dorsum. Rhinophores and tail black: Open parapodia green. Foot sole white with black end.	West Papua, Indonesia	Present study

The original drawings of *Plakobranchus ocellatus* made by van Hasselt (1824) do not show many details (Figure 1), but enough to separate it from *Plakobranchus papua* sp. n. The dorsum and flanks of *Plakobranchus papua* sp. n. have white spots, while *Plakobranchus ocellatus* possesses ocellate spots with dark pigment surrounded by a yellow ring or surrounded by a black ring (Figures 1, 7). These large ocellate spots of *Plakobranchus ocellatus* are also present all over the foot sole, while in *Plakobranchus papua* sp. n. the foot sole is white without any spots. Furthermore, the ocellate spots of *Plakobranchus ocellatus* are very abundant all over the dorsum in combination with broad transverse stripes of darker or lighter pigment. These ocellate spots are abundant on the dorsum of the preserved specimen (Figure 7c, d) and less abundant on the ventral part of the body (Figure 7e). The tail of *Plakobranchus papua* is black, while in *Plakobranchus ocellatus* only the margin of the tail has dark pigment. Bergh (1872) described the radula of *Plakobranchus ocellatus* as consisting of 19 teeth, eight ascendant teeth, one not developed, one tooth at tip and nine descendant teeth, and between 90 to 100 teeth inside the ascus. The teeth possessed up to 14 strong denticles at each side. In *Plakobranchus papua* the shape of the teeth appears more arched than the teeth of *Plakobranchus ocellatus* described and figured by Jensen (1997a). In our specimens of *Plakobranchus papua* the number of teeth is similar, and the lateral denticles ranged between 10 and 14 in number. In the SEM photos of *Plakobranchus ocellatus* by Jensen (1992) at least 10 denticles can be counted. The illustrations presented by Bergh (1873: Plate XI figs 5, 6) for *Plakobranchus chlorophacus* show 10–11 denticles. Within Sacoglossa, species like *Elysia viridis* (Montagu, 1804) are able to modify the teeth size and shape in response to changes in their diet (Jensen 1993) but it is not known if this ability also exists in *Plakobranchus*.

Wägele et al. (2010) discovered special glandular structures on the dorsoanterior to lateral parts of the pharynx in *Plakobranchus ocellatus*. Similar glands were found surrounding the pharynx in *Plakobranchus papua*, and stained dark blue when treated with toluidine blue.

Jensen (1997a) described a pair of copulatory bursae in the reproductive system of *Plakobranchus ocellatus*, which were also found in *Plakobranchus papua* sp. n. (Figure 5: gv).

When comparing the drawings of the *Plakobranchus ocellatus* holotype (Figure 1a–c) and those by Marcus (1982) of specimens collected in the Farasan Islands, Red Sea (Figure 1m, n), differences can be observed between both sets of illustrations. The schematic drawing made by Marcus (1982) is based on one preserved specimen and shows some scattered large ocellated dorsal spots and a line of ocelli along the head (Figure 1m). In the *Plakobranchus ocellatus* described by van Hasselt (1824) the renopericardial area is fused together and appears as a nearly rounded prominence, while in the specimen illustrated by Marcus (1982) the pericardium is separated with respect to the renal prominence and this renal prominence is very elongated (Figure 1n), which is not confirmed in the text (Marcus 1928). Since Red Sea specimens externally resemble *Plakobranchus ocellatus* *s.s.* (Yonow 1990, 2008), these differences need more study before they can be considered reliable. In this regard, it is noteworthy that *Plakobranchus papua* possesses a shorter renopericardial area as that illustrated by van Hasselt for *Plakobranchus ocellatus*.

Marcus (1982) used the absence of dorsal vessels in *Plakobranchus* to separate this genus from *Pattyclaya* (which possesses clear dorsal vessels) and Jensen (1992) also used this same absence to separate *Plakobranchus* from *Elysia*. *Plakobranchus* species have tiny anastomosing lamellae connecting the external lamellae to the most internalones. In *Plakobranchus papua* fine short dorsal anastomosing lamellae connect the anteriormost external lamellae with the most internal ones, which are in direct connection with the renopericardial area. This is endorsed by Wägele et al. (2010), who stated that the Plakobranchidae are characterized as follows: “a number of pericardial vessels are found branching from the pericardium along the dorsal surface of the body and parapodia”.

The new COI sequences were used in a phylogenetic analysis together with the 76 sequences available in GenBank under the name *Plakobranchus ocellatus* from various Indo-West Pacific localities (Figure 8). The phylogenetic analysis shows that *Plakobranchus papua* with *Plakobranchus* sp. 1 from Krug et al. (2013) are in the same cluster, and separated from all other *Plakobranchus ocellatus* sequences. Solely based on COI, *Plakobranchus papua* could be considered the same species as *Plakobranchus* sp. 1 of Krug et al.(2013). This would imply that *Plakobranchus papua* is not restricted to West Papua but also occurs at Panglao Island (Philippines) and Sulawesi (Indonesia).

The ten groupings retrieved in our ABGD analysis and phylogeny reconstruction (Figure 8) agree with the results of Krug et al. (2013). Thus, although morphological analyses indicate a single highly polymorphic *Plakobranchus ocellatus* (Jensen 1992, 1993, 1996, 1997a, 1997b, 2007), molecular analyses indicate a species complex containing ten candidate species. Christa et al. (2013: fig. 1b) identified a specimen from Guam as *Plakobranchus ocellatus*, but according to our *Plakobranchus* colour pattern and distribution review (Table 2), this specimen resembles *Plakobranchus ocellatus* var. A more closely. Unfortunately the GenBank accession numbers cannot be linked to their illustrated specimens. Krug et al. (2013) stated that some differently coloured Japanese specimens could also be considered separate species. The eleven varieties of *Plakobranchus ocellatus* found in the web and publications (one of which is *Plakobranchus papua* sp. n.) could not be linked because Krug et al. (2013) did not provide enough information on the morphology and colouration pattern of their specimens, nor did they include illustrations. Based only on phenotypes of images retrieved from the internet, eleven varieties of *Plakobranchus* were distinguished, including *Plakobranchus ocellatus* (*sensu stricto*) and *Plakobranchus papua* sp. n. (Table 2). All *Plakobranchus* species and varieties were recorded from the Indo-West Pacific, including the Red Sea.

Further research on the genus *Plakobranchus* based on morphology and DNA is necessary to unravel the phylogenetic and taxonomic relationships within this genus, with clear illustrations included of the external morphology. The present study shows that *in situ* photographs are indispensable to morphologically differentiate between *Plakobranchus* species because of their highly variable colour patterns, since ethanol-preserved specimens tend to lose much of their original colouration (Figure 7). Museum collections as a source for taxonomic work, studies on historical ecology, or other kinds of biodiversity studies (Rainbow 2009, Hoeksema et al. 2011, Rocha et al. 2014), may not be sufficient without photographic documentation of the specimens *in situ*. It is suggested that illustrations be included in DNA research as well as included in GenBank data.

## Supplementary Material

XML Treatment for Plakobranchus

XML Treatment for Plakobranchus papua
